# Draft Genome Sequence of Multidrug-Resistant Abortive *Campylobacter jejuni* from Northern California

**DOI:** 10.1128/genomeA.00171-17

**Published:** 2017-04-13

**Authors:** Allison M. Weis, Kristin A. Clothier, Bihua C. Huang, Nguyet Kong, Bart C. Weimer

**Affiliations:** a School of Veterinary Medicine, 100K Pathogen Genome Project, UC Davis, Davis, California, USA; b California Animal Health and Food Safety, Davis, California, USA

## Abstract

*Campylobacter jejuni* is an enteric bacterium that can cause abortion in livestock. This is the release of a multidrug-resistant *Campylobacter jejuni* genome from an isolate that caused an abortion in a cow in northern California. This isolate is part of the 100K Pathogen Genome Project.

## GENOME ANNOUNCEMENT

*Campylobacter jejuni* is an enteric pathogen that affects humans worldwide with infection accompanied by symptoms such as fever and bloody diarrhea, and can lead to autoimmune diseases (1–6). *C. jejuni* is zoonotic, and in livestock it can lead to gastroenteritis and occasionally abortion (7–12). In recent years, several regions in the United States have noted the emergence of an abortive hypervirulent *C. jejuni* isolate (13–15).

Multidrug resistant bacteria are a major worldwide problem, leading to untreatable infection and mortality among humans and other animals (16, 17) that can be transmitted to the human microbiome (18). Here, we describe a multidrug-resistant abortive *C. jejuni* strain from northern California that was isolated from a bovine fetus from a third trimester abortion in 2009 and was uniquely multidrug resistant. This *C. jejuni* genome contains *tetO*, which confers resistance to tetracycline and its derivatives. It also contains *aphA*, a kanamycin resistance gene that is an aminoglycoside phosphotransferase. Lastly, it also contains a Thr-86-Ile mutation in the GyrA protein that results in fluoroquinolone resistance. The *tetO* and *aphA* genes are located on a putative plasmid, whereas *gyrA* is in the chromosome. The genome assembly comprised 54 contigs, 1,708,778 bp, and 1,717 coding sequences, and the sequence depth was 59×. This *C. jejuni* isolate was among a set of animal source isolates that was given to the 100K Pathogen Genome Project (http://www.100kgenomes.org) in the laboratory of Bart Weimer (UC Davis). The isolate was checked for purity (19) and genomic DNA (gDNA) was extracted from culture grown on 5% blood agar plates (UC Davis, VetMed Biological Services) for 1 to 2 days, lysed with an enzyme cocktail (20), purified with a Qiagen QIAamp DNA minikit (51306), and analyzed on the Agilent 2200 TapeStation system with the Genomic DNA ScreenTape assay for integrity of gDNA, as described previously (21–23). Approximately one microgram of gDNA was used for library construction with the KAPA Hyper library preparation kit (Kapa Biosystems, Boston, MA, USA; KK8514), on a PerkinElmer Sciclone NGS Workstation (Waltham, MA, USA). In this process, fragmented double-stranded gDNA molecules were end-repaired (5′), adenylated (3′), and ligated with double stranded DNA adapters (Weimer 384 TS-LT DNA Barcodes, Integrated DNA Technologies, Inc., Coralville, IA, USA), followed by dual-SPRI size selection for targeting a library size of 250 to 500 bp. Final library quality assessment to verify size distribution was performed on a Perkin Elmer Caliper Lab Chip GX using the PerkinElmer Caliper HT 1K kit (CLS701879), and library quantification was completed using a SYBR Green–based qPCR kit and a KAPA library quantification kit (KK4824). Pooled libraries were sequenced on the Illumina HiSeq X TEN platform with a PE150-plus index read at Novogene (Sacramento, CA, USA). Paired-end sequence reads were assembled using ABySS version 1.9.0 (*k*-mer = 64) and annotated with Prokka (24, 25).

### Accession number(s).

This sequence can be found at NCBI SRA BioProject PRJNA186441 under genome SRA accession number SRR5210995, and at NCBI GenBank under the accession number MUJW00000000.
